# Sex susceptibility to ventilator-induced lung injury

**DOI:** 10.1186/s40635-019-0222-9

**Published:** 2019-01-11

**Authors:** Inés López-Alonso, Laura Amado-Rodriguez, Cecilia López-Martínez, Covadonga Huidobro, Guillermo M. Albaiceta

**Affiliations:** 1 Instituto de Investigación Sanitaria del Principado de Asturias, Avda de Roma s/n, 33011 Oviedo, Spain; 2 Centro de investigación biomédica en Red-Enfermedades Respiratorias. Instituto de Salud Carlos III, Madrid, Spain; 3 Departamento de Biología Funcional, Instituto Universitario de Oncología del Principado de Asturias. Universidad de Oviedo, Oviedo, Spain; 4 Unidad de Cuidados Intensivos Cardiológicos, Hospital Universitario Central de Asturias, Oviedo, Spain

To the editor,

Avoidance of ventilator-induced lung injury (VILI), defined as the damage caused by the application of large pressures or volumes to the lung parenchyma, is one of the main objectives of contemporary ventilatory management. This specific form of injury is triggered by a variety of molecular mechanisms involving mechanosensation and mechanotransduction of physical forces, inflammatory responses, activation of intracellular signals, extracellular matrix remodeling, and dysregulation of different forms of cell death. Among these, inflammation plays a key role in the induction of early damage, but also in later repair. In spite of this knowledge, no treatment aimed to reduce VILI based on these mechanisms has been translated into the clinical practice.

The outcome of critically ill ventilated patients depends on the cause of the disease, the specific characteristics of the patient (including comorbidities), and the response to therapies and their consequences. The impact of VILI in outcome falls in this latter category. However, it is not known how previous baseline conditions and applied therapies interact.

Sex is one of the main determinants of health and disease. It has been reported that males show worse intensive care unit (ICU) outcomes [1]. In the specific field of lung injury and mechanical ventilation, females may receive lung protective ventilation less frequently, due to miscalculation of target tidal volumes [2]. Some studies have reported higher incidence of acute respiratory distress syndrome (ARDS) [3] and mortality rates [4] and worse long-term outcomes [5] in mechanically ventilated women, but others have failed to confirm these results [6, 7]. The mechanisms behind these differences have been addressed in several experimental studies (reviewed in [8]). Male and female animals have different responses in models of respiratory diseases such as chronic obstructive pulmonary disease (COPD), asthma, and fibrosis. The effects of sex hormones on inflammation and cell metabolism mediate the majority of these differences.

The role of VILI in the difference in ICU outcome between sexes has not been addressed. Most of the experimental research has been conducted in male animals, discarding their female counterparts, and no study has compared male and female animals after mechanical ventilation. To address this question, we ventilated male and female C57BL/6 mice to induce a moderate lung injury (peak pressure 15 cmH_2_O, PEEP 0 cmH_2_O, respiratory rate 100 breaths/minute, inspiratory to expiratory ratio 1:1, FiO_2_ 0.21). After 2.5 h, animals were sacrificed and the lungs removed and fixed. There were no differences in lung injury, addressed using a semiquantitative score [9] (0.5 ± 0.5 vs 0.75 ± 0.52 for female and male animals respectively at baseline, 2.3 ± 1.2 vs 2.2 ± 1.3 for female and male animals respectively after VILI, *n* = 6 per condition, *p* < 0.001 for the effect of ventilation, *p* = 0.99 for the effect of sex in a two-way ANOVA). Using a more aggressive ventilatory strategy (peak pressure 20 cmH_2_O, PEEP 0 cmH_2_O, respiratory rate 50 breaths/minute, inspiratory to expiratory ratio 1:1, FiO_2_ 0.21) yielded slightly higher scores but again without differences between sexes (2.5 ± 1.3 vs 2.7 ± 1.3 for female and male mice, *n* = 3 per group, *p* = 0.88). Similarly, there were no differences in neutrophilic infiltrates between sexes (14 ± 7 vs 11 ± 5 myeloperoxidase-positive cells per field in female and male animals respectively at baseline, 43 ± 19 and 37 ± 15 myeloperoxidase-positive cells per field in female and male animals respectively after ventilation, *n* = 6 per group, *p* = 0.001 for the effect of ventilation, *p* = 0.80 for the effect of sex). Figure 1 shows representative sections of these studies.

**Fig. 1:**
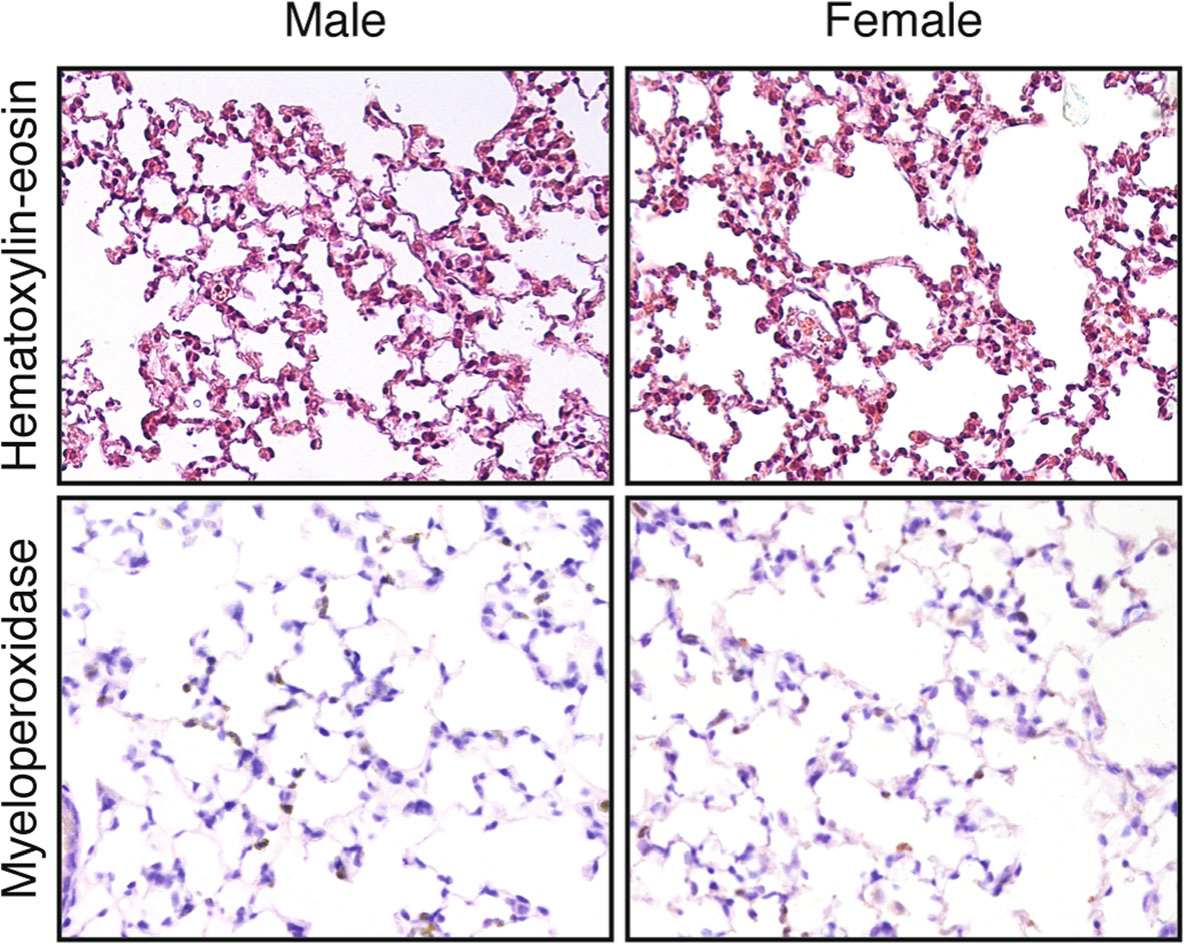
Representative histological sections stained with hematoxylin-eosin (upper row) or immunostained using an anti-myeloperoxidase (MPO) antibody (lower row) in male and female mice after injurious ventilation

In order to obtain a large-scale assessment of the putative differences between groups, RNA from lung homogenates from baseline and ventilated mice of each sex was obtained and hybridized using expression microarrays (Affymetrix Mouse Gene 2.0 ST, with a coverage above 26,500 genes). Raw data (available at GEO database, accession number GSE121550) were background-corrected and normalized to obtain expression values. These values were fitted to a linear model including sex and ventilation as factors, and *F* statistics computed with Bayesian moderation of the standard errors. *p* values were corrected using the Benjamini-Hochberg method. These methods have been detailed elsewhere [9]. Mechanical ventilation induced significant changes in 3510 genes, irrespective of the sex. When comparing the effect of mechanical ventilation between sexes, only 30 genes showed a log-fold change higher than 1.5 (this is, a 2.8-fold difference between male and female above in the ventilator-induced change in gene expression). Irrespective of the fold change, no gene showed a significant difference between sexes reaching an adjusted *p* value lower than 0.05 (Fig. 2).

**Fig. 2:**
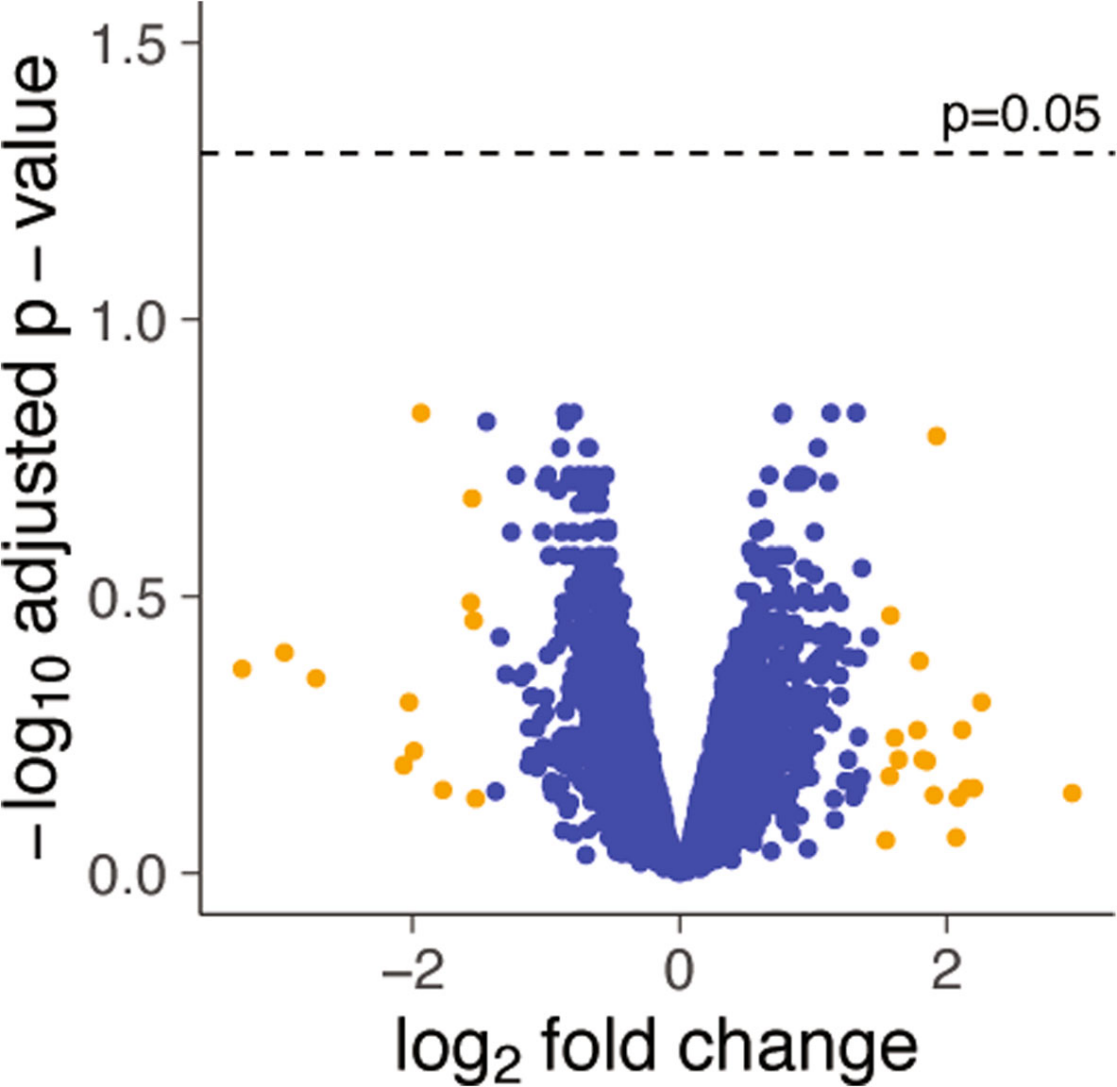
Volcano plot representing the log-fold change in gene expression with ventilation (comparing male and female mice) against the log of the statistical significance. Only 30 genes (orange dots) had a log-fold change above 1.5, and no gene reached an adjusted *p* value lower than 0.05

Collectively, these results suggest that there are no differences caused by sex in susceptibility to VILI. Rather than justify the use of animals of a single sex in experimental studies, these findings support the inclusion of both, as advocated by others [10]. Our data allow researchers to assume a hypothesis of no influence of sex, followed by the post hoc assessment of putative differences, if any.

## Abbreviations

ARDS: Acute respiratory distress syndrome
COPD: Chronic obstructive pulmonary disease
ICU: Intensive care unit
VILI: Ventilator-induced lung injury
